# The effect of Nullomer-derived peptides 9R, 9S1R and 124R on the NCI-60 panel and normal cell lines

**DOI:** 10.1186/s12885-017-3514-z

**Published:** 2017-08-09

**Authors:** Abdelkrim Alileche, Greg Hampikian

**Affiliations:** Biology Department Room SN-215, Boise State University, 1910 University Drive, Boise, ID 83725 USA

**Keywords:** Cancer, Peptides, Metabolism, ATP depletion, NCI-60, Nullomers

## Abstract

**Background:**

Nullomer peptides are the smallest sequences absent from databases of natural proteins. We first began compiling a list of absent 5-amino acid strings in 2006 (1). We report here the effects of Nullomer-derived peptides 9R, 9S1R and 124R on the NCI-60 panel, derived from human cancers of 9 organs (kidney, ovary, skin melanoma, lung, brain, lung, colon, prostate and the hematopoietic system), and four normal cell lines (endothelial HUVEC, skin fibroblasts BJ, colon epithelial FHC and normal prostate RWPE-1).

**Methods:**

NCI-60 cancer cell panel and four normal cell lines were cultured in vitro in RPMI1640 supplemented with 10% Hyclone fetal bovine serum and exposed for 48 h to 5 μM, 25 μM and 50 μM of peptides 9R, 9S1R and 124R. Viability was assessed by CCK-8 assay. For peptide ATP depletion effects, one cell line representing each organ in the NCI-60 panel, and four normal cell lines were exposed to 50 μM of peptides 9R, 9S1R and 124R for 3 h. The ATP content was assessed in whole cells, and their supernatants.

**Results:**

Peptides 9S1R and 9R are respectively lethal to 95 and 81.6% of the 60 cancer cell lines tested. Control peptide 124R has no effect on the growth of these cells. Especially interesting the fact that peptides 9R and 9S1R are capable of killing drug-resistant and hormone-resistant cell lines, and even cancer stem cells. Peptides 9R and 9S1R have a broader activity spectrum than many cancer drugs in current use, can completely deplete cellular ATP within 3 h, and are less toxic to 3 of the 4 normal cell lines tested than they are to several cancers.

**Conclusions:**

Nullomer peptides 9R and 9S1R have a large broad lethal effect on cancer cell lines derived from nine organs represented in the NCI-60 panel. This broad activity crosses many of the categorical divisions used in the general classification of cancers: solid vs liquid cancers, drug sensitive vs drug resistant, hormone sensitive vs hormone resistant, cytokine sensitive vs cytokine non sensitive, slow growing vs rapid growing, differentiated vs dedifferentiated cancers. Furthermore peptides 9R and 9S1R are lethal to cancer stem cells and breast canrcinosarcoma.

**Electronic supplementary material:**

The online version of this article (doi:10.1186/s12885-017-3514-z) contains supplementary material, which is available to authorized users.

## Background

Nullomers are the shortest string of monomers absent from a species or species group. In 2007 Hampikian and Andersen [1] published a list of Nullomers that were absent from all publicly available databases (called Primes); for peptides there were 198 (5-amino acid strings) absent from all known proteins. Nullomer DNA sequences have been used to “watermark” DNA samples and prevent forensic contamination [2], and Nullomer-derived peptides have been shown to be effective against prostate and breast cancer cell lines [3]. In this study, we examined the effects of 3 Nullomer-derived peptides on the NCI-60 panel of cancer cells, and 4 normal cell lines.

The current treatment of cancer cannot address many aspects of human tumors driven by their cellular heterogeneity. For example, some cells in a tumor may be designated drug resistant [4] cancer cells, but other types of cells, such as quiescent tumor cells and cancer stem cells [5], are also out of reach of many of the current drug protocols. Due to these challenges, a new treatment paradigm is emerging based on the targeting cancer cell metabolism, including aerobic glycolysis (Warburg effect). We report here that two Nullomer-derived peptides (9R, 9S1R) have a broad spectrum of activity against the NCI-60 panel, and lead to dramatic cellular ATP depletion.

## Methods

### Cell lines

The NCI-60 panel of cancer cells was purchased from Development Therapeutics of the NCI. Cells were thawed in a water bath at 37 °C, and collected by centrifugation at room temperature. For maintenance, the cell were grown in RPMI 1640 supplemented with 10% Hyclone fetal bovine serum (FBS), and 1× penicillin streptomycin (100× solution, Invitrogen) at 37 °C and 5% CO_2_. Passaging of cells was done at 80% confluence after a PBS wash, cells were treated with Trypsin-EDTA 0.25% (Invitrogen).

Normal cell lines were purchased from the American Type Culture Collection (ATCC, Manassas, VA). Human endothelial cell line HUVEC (ATCC CRL-1730) was grown in the ATCC formulated F-12 K medium with 0.1 mg/ml Heparin, 0.05 mg/ml endothelial cell growth supplement and 10% FBS. Human skin fibroblast BJ (ATCC CRL-2522) was grown in the ATCC formulated Eagle’s Minimum Essential Medium supplemented with 10% FBS. Human prostate epithelial cell line RWPE-1 (ATCC CRL-11609) was grown in the Keratinocyte Serum Free Medium (K-SFM, GIBCO) supplemented with 0.05 mg/ml bovine pituitary extract (BPE) and 5 ng/ml human recombinant epidermal growth factor (EGF). Human colon epithelial cell line FHC (ATCC CRL-1831) was grown in the ATCC formulated DMEM-F12 supplemented with 10 mM HEPES (final concentration 25 mM), 10 ng/ml cholera toxin, 0.005 mg/ml insulin, 0.005 mg/ml transferrin, 100 ng/ml hydrocortisone and 10% FBS. Only early cell passages were used for all experiments. No human subjects (including human material or human data) has been used in the research reported in this manuscript. All cancer ell lines were obtained from the NCI depository, and all normal cells from the ATCC.

### Reagents

Peptides 9R, 9S1R and 124R were made by Pierce Biotechnology (Rockford, IL). HPLC purified peptides (purity >98%) were delivered in 0.5 mg/tube (lyophilized) and stored at −20 °C. Peptides were solubilized in 1 M Trehalose to produce 50 mM stock solutions. Only freshly prepared solutions were used to treat cells. Trehalose final concentration in all reactions was 1 mM (a dose without any effect on the growth of cancer and normal cells). Sodium Azide (Sigma Aldrich) was solubilized in water at 100 mM.

### Screening the effect of peptides 9R, 9S1R and 124R on the NCI-60 panel and normal cells

Three thousand–Five thousand normal or cancer cells/well were seeded in 96-well plates. After 24 h incubation, peptides 9R, 9S1R and 124R were added to the wells, with untreated cells as a control. After 48 h exposure to 5 μM, 25 μM, or 50 μM peptides, cell viability was quantified by the addition of 10 μl of cell counting kit (CCK-8, Dojindo Japan) to each well, incubated for 4 h at 37 °C in a 5% CO_2_ incubator. After the 4 h incubation, plates were monitored by a plate reader (SynergyMx from BioTek, Winooski, VT) at an absorbance of 450 nm. Plate readings were exported to Microsoft Excel and GraphPadPrism software. All the wells were analyzed in triplicates. The statistical analysis was done with GraphPadPrism*. The survival index (SI) is the CCK-8 reading ratio of peptide-treated to untreated cells (minus the blank values), multiplied by 100 to yield an index of the percent surviving. A dose response using several concentrations of the peptides (5 μM, 25 μM and 50 μM) was used for all cancer and normal cell lines. The SI was used for screening in this study at the 50 μM concentration of each peptide.

### Measurement of cellular and cellular-supernatant ATP content

Cancer cell lines (one cell line for each organ represented in the NCI-60 panel) and normal cell lines (3000-5000/well) were seeded in 96 well microplates (white plates from Nunc to block luminescence bleeding between the wells), and allowed to attach for 24 h. Then cells were incubated for 3 h with peptides (50μΜ of 9R, 9S1R and 124R), or 100 mM sodium azide as a control. After 3 h, 10 μl of a single reagent from Cell Titer Glow™ (Promega) was added to cells. For the cellular supernatant ATP content, the supernatant of each well was transferred to a new microplate and 10 μl of a single reagent from Cell Titer Glow™ (Promega) was added to each supernatant. Complete reagent mixing in 96 wells plates required gentle orbital shaking for 2–10 min. The plate reading was taken by a SynergyMx plate reader. Plate readings were exported to Microsoft Excel and Graph-PadPrism software. All the wells were analyzed in triplicates. The statistical analysis was done with the GraphPadPRISM*.

## Results

### NCI-60 cancer cell lines’ sensitivity to peptides 9R, 9S1R and 124R

In a previous work [3] peptide 9R (RRRRRNWMWC) and its scrambled version 9S1R (RRRRRWCMNW) were shown to be lethal to LnCap (prostate), MDA MB 231 (breast), B16 (mouse melanoma), and HUT 102 (Adult T cell leukemia) cancer cell lines; while having more limited lethal effects on normal cell lines HMEC (breast), PCS (prostate), WI-38 (fibroblast), and J774A.1 (mouse macrophages). Peptide 124R, a Nullomer-derived control peptide (RRRRRWFMHW) had no effect on any of the cells tested. In the present study, we extended the Nullomer anticancer investigation to the full NCI-60 panel [6], which includes cancer cell lines derived from a wide variety of human tumors originating from nine organs. These tumors represent many types and stages of cancer evolution in regard to both their metastatic potential, and sensitivity or resistance to drugs.

The three peptides were prepared as previously published: first solubilized in 1 M Trehalose and then diluted to three doses, 50 μM, 25 μM, and 5 μM final concentration. Cells were incubated for 48 h, and then their viability was assessed by CCK-8 assay [7]. Cell lines were considered sensitive to peptides 9R, 9S1R and 124R if the survival index (SI) was less than 80%. This means that for sensitive cell lines at least 20% of the cells were killed by 50 μM peptide in a 48 h exposure.

As shown in [“Additional file 1: Results S1” and “Additional file 2: Table S1” (A, B)] peptides 9R and 9S1R have broad activity against the NCI-60 panel: 95% of the cancers are sensitive to peptide 9S1R, and 81.6% are sensitive to peptide 9R; all of cell lines tested are resistant to peptide 124R. Fifty seven cell lines are sensitive to 50μΜ of 9S1R, with a SI between 3.80% (MALME-3 M) and 71.20% (MOLT-4). Notably, nine cell lines have a SI of less than 10%: kidney UO-31 and ACHN, prostate DU-145, ovarian OVCAR-8 and OVCAR-4, colon COLO 205, melanoma UACC-257 and SK-MEL-5 and MALME-3 M, and breast HS-578 T. Only three cell lines (5%) are resistant to peptide 9S1R: colon cancer HT-29 and HCC 2998, and lung cancer A-549 ATCC.

Forty-nine of the NCI-60 cancer lines are sensitive to peptide 9R with a SI between 4.10% (MALME-3 M) and 78.10% (IGR-OV1). Four cell lines have a SI of less than 10% after exposure to 9R: melanoma SK-MEL-5 and MALME-3 M, breast HS-578 T, and lung EKVX. Eleven cell lines (18.3%) are resistant to peptide 9R: kidney A498 and UO-31; prostate PC-3; ovarian SK-OV-3; colon HT-29; melanoma SK-MEL-28; breast MCF-7; lung NCI-H226, NCH-H522, A549 ATCC and NCI-H322M. All told, peptide 9R and 9S1R are lethal to cancer cells from all nine organs represented in the NCI-60 panel.

We have classified each cell line according to the four possible combinations of Nullomer sensitivity response [“Additional file 2: Table S1” (C)]. Eighty percent of the NCI-60 cancer cell lines are sensitive to both peptides 9R and 9S1R (category I). For example, all the leukemic and CNS cell lines are sensitive to both peptides 9R and 9S1R and thus belong to category I. At the opposite end of the spectrum, two cell lines are resistant to both peptides 9R and 9S1R (category IV): colon HT-29 and lung A549 ATCC. Fifteen percent of the NCI-60 lines are sensitive to 9S1R, but resistant to 9R (category II). Only one cell line, colon HCC 2998, is sensitive to 9R but resistant to 9S1R (category III). Since peptides 9R and 9S1R are scrambled versions of each other, the difference in sensitivities indicates that they likely have different targets within the cell.

It is notable that both peptides 9R and 9S1R kill prostate cancers that are androgen independent (PC-3 and DU-145), and those that are androgen dependent for their growth (LnCap) [3]. Furthermore, peptides 9R and 9S1R kill breast cancer cell lines that are hormone independent and triple negative (MDA-MB-231, BT-549, HS-578 T, and MDA-MB-468), as well as those that are hormone dependent (MCF-7 and T-47D). The killing effect of peptides 9R and 9S1R is not limited to any particular range of doubling time (DT) [(http://dtp.nci.nih.gov/docs/misc/common_files/cell_list.html), “Additional file 2: Table S1”, (A)]. Cells with low DT like colon HCT-116 (17.4 h) and K-562 (19.6 h), as well as those with high DT like lung HOP-92 (79.5 h) and CNS SNB-75 (62.8 h), are all killed by peptides 9R and 9S1R. Since our screening was limited to 48 h peptide exposure, cell lines with high DT didn’t have time to complete a full cell cycle. This implies that the targets for peptides 9R and 9S1R are not involved in DNA replication or mitosis.

We have compared the activity spectrum of peptides 9R and 9S1R with ten classes of anticancer drugs approved by the FDA for patients ([8], “Additional file 3: Table S2”), most of which are used in combination. There is no single anticancer drug that matches the broad activity spectrum of peptides 9R and 9S1R against cancers from nine organs, including solid tumors (like prostate, breast, CNS and lung cancers), as well as liquid tumors (leukemia, lymphoma). Although many classes of anticancer drugs target DNA replication or microtubules, these drugs do not have the same broad spectrum of activity as 9R and 9S1R. We note that CNS, kidney, colon and melanoma cancers have fewer available drug treatment options, compared to leukemia, ovarian and breast cancers--but peptides 9R and 9S1R are active against all of these.

### Sensitivity of normal cells to peptides 9R, 9S1R and 124R in viability assays

Normal cells from four organs: skin fibroblasts BJ, endothelium HUVEC, colon epithelium FHC, and prostate RWPE-1 cell lines were also treated with 9R and 9S1R [“Additional file 4: Results S2” and “Additional file 2: Table S1”, D)]. At 25 μM, 9R has no effect on HUVEC or BJ normal cell lines; and the effect on the constantly-renewing intestinal cell line (FHC) was below our sensitivity threshold of 20% lethality (the cells were 90% viable). Of the non-cancer cells tested, RWPE-1 prostate cells showed the greatest sensitivity to 9R, dropping just below our sensitivity threshold of 20%, (the cells were 79% viable). However, at this dose many cancer cell lines in our screen suffer far greater lethality. For example, treatment with 25 μM 9R decreases viability of several cancers by over 50%: M14 melanoma, EKVX lung cancer, HCC 2998 colon cancer, SR and CCRF leukemia cell lines. Future studies will determine the most effective doses for each peptide against each particular cancer cell line.

Sensitivity of normal cells to 9S1R was greater than that of 9R, however two of the four normal cell lines tested showed no effect on viability at 25 μM 9S1R (HUVEC and BJ). FHC cells treated with 25 μM 9S1R had 26% reduced viability (74% survived), and RWPE-1 cells’ viability was reduced by 79% (21% survived).

The present study is principally a screening assessment of 9R and 9S1R with results shown for three doses (50 μM, 25 μM and 5 μM). We have analyzed and classified the results for all 64 cell lines based on reaction to the highest dose (50 μM), however, we expect that modifications to these peptides will allow for lower clinical doses. The differential response of various cancer lines, and the relative resistance of some normal cell lines, points to both the promise and challenges for translation of these Nullomer peptide drugs to the clinical setting. The promise of these peptides is perhaps best illustrated by the fact that doses of 25 μM 9R are effective against several cancer cell lines (see Additional file 1: Result S1), but have little or no effect on normal cells.

### Peptides 9R and 9S1R ATP depletion effect on cancer cells

**Fig. 1:**
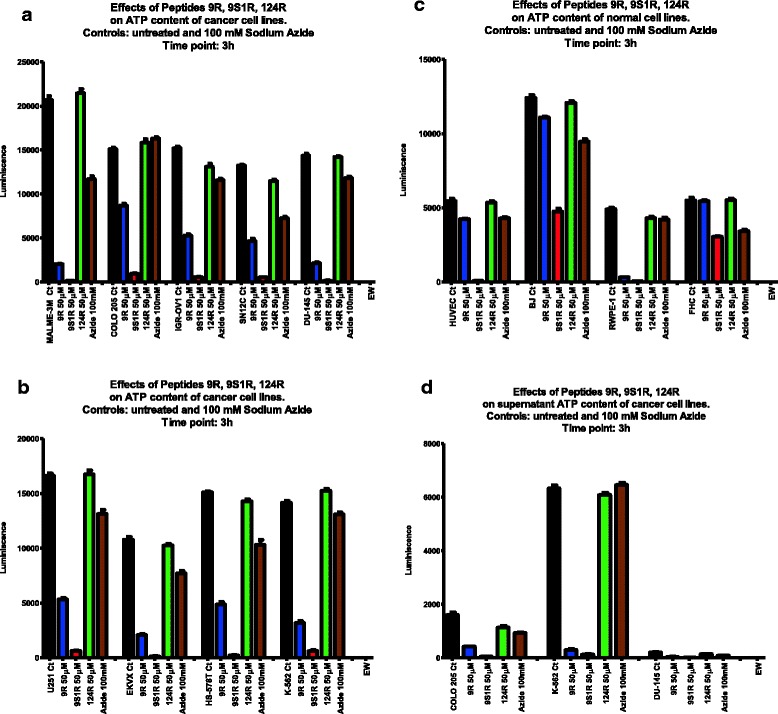
ATP depletion of cancer and normal cell lines by Peptides 9R, 9S1R, 124R and sodium azide. 50 μM of peptides 9R, 9S1R and 124R (3 h incubation) effects on cellular ATP content of cancer and normal cell lines. **a**: MALME3M, COLO205, IGR-OV1, SN12C and DU-145; **b**: U251, EKVX, HS-578 T and K-562; **c**: HUVEC, BJ, RWPE-1 and FHC; **d**: Supernatant ATP content of cancer cells COLO205, K-562 and DU-145. Controls: untreated cells, 100 mM Sodium Azide, EW (empty wells). All wells in triplicates. Each panel is a representative of three experiments

We examined the cellular ATP content for one line from each organ represented in the NCI-60 panel: SN12C, DU-145, IGR-OV 1, K-562, COLO 205, MALM-3 M, HS-578 T, U251 and EKVX. Cellular ATP content was evaluated after 3 h exposure to 50μΜ of peptides 9R, 9S1R and 124R. In just 3 h, 9S1R reduced the cellular ATP content of all nine cell lines by more than 90%, resulting in ATP levels that were a small fraction of the untreated cells: from 0.59% (MALM-3 M) to 6.65% (COLO 205) [Fig. 1 (a, b), “Additional file 5: Table S3”]. The ATP depletion effect of peptide 9R is also substantial, reducing ATP to between 9.87% (MALM-3 M) and 58.56% (COLO 205) of the untreated cells. This difference between the ATP depletion effect of peptides 9R and 9S1R may be due to the differential expression of different cellular targets in these cells. For comparison, we also treated each cell line with 100 mM sodium azide, a selective inhibitor of complex IV in the mitochondrial respiratory chain. In many cases, the two peptides had a greater ATP depletion effect (measured after 3 h) than the 100 mM sodium azide.

### Peptides 9R and 9S1R ATP depletion effect on normal cells

Normal cell lines (HUVEC, BJ, FHC and RWPE-1) were tested in the same way as the cancer cells. At 50 μM peptide 9S1R completely depleted the cellular ATP content of HUVEC and RWPE-1 cells; however, its effect on BJ and FHC cells was moderate. In fact, while 9S1R reduced cellular ATP in all the cancer lines below 7% of the untreated cells, ATP in 2 of 4 normal cell lines was 39% of the untreated (BJ), and 54% (FHC) of the untreated. We note that depletion of ATP was not directly related to loss of vitality for all cell lines. This differential response to 9S1R-even at high dosage-suggests that this peptide may be useful to treat specific tumors.

Peptide 9R completely depleted the ATP content of RWPE-1, but had no effect on the three other normal cell lines. Peptide 124R produced no ATP depletion effect on normal cells [Fig. 1 (c), “Additional file 5: Table S3”], and served as an effective control Nullomer-derived peptide treatment.

### Peptides 9R, 9S1R and 124R effects on ATP levels in cellular supernatants

F_0_F_1_-ATP Synthase is normally expressed by the internal membrane of the mitochondria, but many cancer cells express it on the cell surface where it secretes ATP into the cell supernatant [9]. In order to explore ATP production in greater detail, we treated cancer cells with the three peptides, and determined the ATP content in the supernatants after 3 h’s incubation. As shown in Fig. 1 (d), peptides 9R and 9S1R deplete the ATP secreted by K-562 and COLO 205 cells into the culture supernatant. While the significance of this effect needs further exploration, one possible target of the Nullomer-derived peptides may be Ectopic F_0_F_1_-ATP Synthase.

## Discussion

### Peptides 9R and 9S1R are COMPARE-negative drugs: Affecting a great variety of cancer cell types

To our knowledge, this is the first study of the anticancer effects of synthetic small peptides on the NCI-60 panel. The website http://crdd.osdd.net/raghava/cancerppd/ [10] reviews 3491 anticancer peptides (ACP), but none have been tested against the full NCI-60 cancer cell panel in a single study. Since its introduction in 1990 the *“NCI-60 screen”* [6] failed to identify any single drug with broad activity against cancer types. Although there are specific markers for many cancers, the uncontrolled growth of tumors is the most obvious trait shared by all of them, along with local invasion and distant metastasis. One might think that any drug capable of inhibiting DNA replication or the mitotic apparatus would be effective against many types of cancers. However, the anticancer drugs approved by the FDA, described in “*Cancer: Principles & Practice of Oncology*” [8], do not include a single drug used in the treatment of all nine tissue types represented in the NCI-60 panel. This is also true for all the drugs tested against the larger cancer cell line panels, for example the cancer cell line encyclopedia (CCLE), which includes 1036 cell lines [11], and the cancer genome project (CGP) with 727 cell lines [12]. Each approved anticancer drug has a tumor-type selectivity or “*fingerprint”* that can be analyzed using the “*COMPARE algorithm*” [13]. Since peptides 9R and 9S1R are active against cancers from all nine organs represented in the NCI-60 panel, they can be considered “*COMPARE-negative drugs*” and this makes them different from other tested drugs.

It is remarkable that cancer cells from all nine NCI-60 organs (kidney, prostate, ovary, hematopoietic system, skin, CNS, colon, and lung) are sensitive to 9R and 9S1R peptides. These nine organs differ widely in their functions, metabolism, and reproductive rates. The skin and the hematopoietic system constitutively renew, while the ovary and the brain are nearly quiescent. Of course all of the cancer lines share some basic physiological mechanisms like the production of ATP by glycolysis and/or oxidative phosphorylation. Peptide 9R and 9S1R’s broad spectrum of activity can be explained by some common feature of all types of tissues represented in the NCI-60 panel--a feature that we expect will be found in what is called “*cells of origin of cancer”* [14]. Peptide 9R and 9S1R defy many of the commonly used classification systems for cancer drug types, and further studies are needed to more fully characterize and classify these drugs.

### Histology types affected by Nullomer-derived peptides 9R and 9S1R

The NCI-60 panel includes a wide array of histological types (http://discover.nci.nih.gov/cellminer/celllineMetadata.do). We show that peptide 9R and 9S1R sensitivity is not restricted to any particular histological type in the panel. This is important since cancer generally evolves from histologically distinct and differentiated cells, to an undifferentiated state that is characterized by local and distant metastasis with accompanying drug resistance. Peptides 9R and 9S1R are effective against undifferentiated glioblastomas (SF-268, SF-295, SF-539, U251); undifferentiated lung cancers (HOP-62, HOP-92, NCI-H460); poorly differentiated ovarian cancer (IGR-OV1), moderately differentiated ovarian cancers (OVCAR-3, OVCAR-4) and well differentiated ovarian cancer (OVCAR-5); poorly differentiated kidney cancers (SN-12C, RXF 393); amelanotic melanoma (LOX IMVI) and melanotic melanoma (SK-ML-2, M14, UACC-62, UACC-257). In addition, peptides 9R and 9S1R are effective against adenocarcinomas (cancers of glandular tissues) like colon HCC-2998 and breast MCF-7, carcinomas (cancers from epithelial tissues) like lung NCI-H226, and blastomas (cancers from embryonic tissue of organs) like CNS SF-268. Furthermore, the breast cancer cell line HS578T is sensitive to both peptides 9R and 9S1R. This type of breast cancer, which constitutes 1% of all breast cancers, is a complex combination of epithelial and mesenchymal metaplasic carcinoma (carcinosarcoma) [15]. Such cancers are far more aggressive and have a poorer prognosis than even triple negative breast cancers.

### Peptide 9R and 9S1R effects on solid vs liquid tumors

The treatment of solid and liquid cancers in patients is quite different from cell culture conditions. However, one can assess the effects of novel drugs on tumors isolated from both solid and liquid tissues in culture. Cancers of the ovary, CNS, lung, kidney, melanoma, prostate, colon and breast are considered solid tumors. Leukemia and myeloma are considered liquid cancers. Normally cancer drug effects are limited to solid or leukemic cancers [8]. For example, taxol and its derivatives are not effective against leukemia, and the proteasome inhibitor Velcade has been used effectively only against myeloma. In general, leukemia has a better prognosis than solid tumors.

As shown in “Additional file 3: Table S2” there are 29 drugs currently used against leukemia, 17 drugs against breast cancers, 7 drugs against melanoma, 8 drugs against kidney cancers, and only 5 drugs against CNS cancers. Peptides 9R and 9S1R are effective against all of these cancer cells lines (both solid and liquid cancers) (“Additional file 2: Table S1”), including the aggressive ATL leukemia (HUT102) [3]. Therefore the target (s) of peptides 9R and 9S1R are expressed by solid and liquid tumors.

### The doubling time (DT) of the NCI-60 cancer cell lines panel

The diversity of cancer cell line DTs has practical implications for cancer research. It is difficult to standardize all the parameters of in vitro cell growth (cell density, degree of confluency, volume of culture medium, types of 96-1536 wells microplates, and the exposure time to drugs), especially when using large panels of cell lines that have varying DTs. Because of this, the literature is replete with varying screening protocols. For example, the NCI 48-h incubation protocol for the NCI-60 screen uses a scale of inoculation densities to accommodate various DTs (5000–40,000 cells per well in a 96 well microplate) [6]. For a panel of 639 cancer cell lines Garnett et al. [10] used cell densities of 70% confluency in 96 and 384 wells microplates, with a 72 h exposure to drugs. With the availability of automated platforms, Griner et al. [16] used 1000 cells/5 μl in a 1536 well plate, and a 48 h exposure to drugs. In our screening method, we used 5000 cells/100 μl in 96 wells plate and an exposure time of 48 h. These differences in research protocols make it difficult to compare results across studies. Nevertheless, we have shown that Nullomer-derived peptides 9R and 9S1R are effective against cancer cell lines with short (~24 h) and long (62-79.5 h) DTs. For example, the peptides were effective against kidney cancer lines 786.0 (22.4 h DT) and RFX 393 (62.9 h DT); CNS cancers U251 (23.8 h) and SNB-75 (62.8 h), lung cancers NCI-H460 (17.8 h) and HOP 92 (79.5 h). Since we used a treatment period less than the DT of many NCI-60 cells, and the peptides were effective against cells having a broad range of DTs, peptides 9R and 9S1R do not appear to target DNA replication or the mitotic apparatus.

### Peptides 9R and 9S1R kill resistant and non-resistant cancer cells

In the context of this study, cancer cell resistance is in fact four distinct phenomena: resistance to drugs, hormone and cytokine resistance, resistance of cancer stem cells, and peptide resistance. It would be misleading to put them all in the same category [17], and we separate them here.

#### Drug resistance

Resistance starts with one drug, but often evolves to what is called multidrug resistance (MDR), usually mediated by the ABC transporter family. A review by Szakacs et al. [18] shows that among 22 clinical trials (two of which are not yet finished), 15 of 20 drugs deliver “no benefit” to patients due to secondary resistance. In that review, he proposes that, “*drug resistance can be reversed by hydrophobic peptides*”. In keeping with that suggestion, we have tested the Nullomer-derived peptides against all 60 lines in the NCI-60 panel. In our preparation for this study, we found that identifying which cell lines are “drug resistant” among the NCI-60 panel is not a trivial matter of literature search. In fact, we have found much contradictory information in different published works--just as Baggerly et al. documented in their seminal study [19]. We finally based our own evaluation criteria for drug resistance on the work by Yadav et al. [20], in which they investigated the sensitivity of the NCI-60 panel to 1400 drugs, and established a list of drug resistant and drug sensitive cell lines. From the Yadav et al. list of drug resistant cell lines we found several that are sensitive to peptide 9S1R: NCI/ADR-RES, SK-OV-3, OVCAR-5, HS-578 T, MDA-MB-231, SNB-19, EKCX, NCI-H460, HOP 62, NCI-H322M. Of the Yadav classified drug resistant lines that are sensitive to 9S1R, with the exception SK-OV-3 and NCI-H322M, all are also sensitive to 9R**.**

#### Hormone and cytokine resistance

Breast, prostate and ovarian cancers have a hormone dependency phase. In this way, the hormone resistance phenotype is different from MDR. Peptide 9R and 9S1R kill both hormone sensitive and hormone resistant cancers. For example, both peptides kill hormone sensitive prostate cancers such as LnCap [3], and hormone resistant prostate cancers like PC-3 and DU-145 (“Additional file 2: Table S1”**).** For breast cancer, peptide 9S1R kills hormone resistant breast cancers (MDA-MB-231, BT-549, HS 578 T and MDA-MB-468), and hormone sensitive breast cancers (MCF-7 and T-47D) (“Additional file 2: Table S1”**)**. Peptide 9R kills all the same lines with the exception of the MCF-7 cell line (“Additional file 2: Table S1”**).** Cytokines, like interleukin 2, are growth factors necessary for the survival and growth of many immune cells. Both peptides 9R and 9S1R kill both interleukin 2-dependent leukemia MOLT-4 ([21], Suppl. “Additional file 2: Table S1”**)**, and the interleukin 2-independent HUT 102 [3].

#### Cancer stem cells resistance

Cancer stem cells play an important role in cancer relapse, whether the stem cells have their origin in the initial tumor, or are induced by chemotherapy. They have been described in solid tumors and hematopoietic malignancies. These cells are characterized by their resistance to drugs [22] and radiation. Both peptides 9R and 9S1R kill cancer stem cells like Ovarian OVCAR-3 [23], lung cancer NCI-H460 [24], and colon cancer HCT116 ([25], “Additional file 2: Table S1”**)**.

#### Peptide resistance

Resistance to peptides is a new phenomenon in the literature because peptides have been considered *“resistance free”* [26]. We have found resistance to our peptide drugs to be cell line specific. Cancer cell line A549 ATCC is drug resistant in the Yadav et al. list [20], and is resistant to both peptides 9R and 9S1R. Two other cell lines (NCI-H322M and SK-OV-3) are resistant to drugs in the Yadav et al. list [20], and are resistant to peptide 9R--but they are sensitive to 9S1R.

### Peptides 9R and 9S1R target tumor heterogeneity

Tumors are heterogeneous in their composition [27] and include non-cancer cells: cells of the stroma such as macrophages, tumor-associated fibroblasts, and endothelial cells. Tumor *genetic* heterogeneity is another dimension of their complexity that can promote resistance, with cells from a single tumor having different genetic makeup and therefore different responses to treatment. Currently, genetic variation is the main reason for chemo resistance in vivo [28]. We have tested the Nullomer-derived peptides 9R and 9S1R against the genetically heterogeneous NCI-60 panel representing nine organs from 60 different donors. The peptides proved effective against a wide variety of cell and genetic types: poorly differentiated and differentiated cancers, solid tumors and cancers of the hematopoietic system, drug resistant and drug sensitive cancer cells, hormone dependent and hormone resistant cancers, cancer stem cells, endothelial cells and macrophages [3], a carcinosarcoma cell line, actively dividing cancer cells and those with very slow growth.

### The ATP depletion effect of peptides 9R and 9S1R

The ATP depletion effect of peptide 9S1R is rapid, almost as rapid as the anthrax lethal toxin [29], and much faster than the effect of 100 mM of sodium azide [30]. Cancer cell metabolism is often dependent on glycolysis for its ATP (the *“Warburg effect”).* However, there is a large variation in the relative contributions of glycolysis and oxidative phosphorylation (Kreb’s cycle, the respiratory chain and the F_0_F_1_-ATP Synthase) in the ATP production of different cancer cells [31]. According to Zu et al.*,* the ATP produced by glycolysis can represent between 0.32and 64% of the cellular total [32]. Therefore, the ATP depletion effect of the glycolysis inhibitor [3-bromo pyruvate (3-BP)] or oligomycin (specific inhibitor of the proton channel F_0_ subunit of the mitochondrial F_0_F_1_-ATPase) depends on the relative contribution of mitochondrial versus glycolytic ATP production [33]. In different cancer cell lines, 3-BP can totally deplete cellular ATP [34] or have no effect at all [35]. In the same vein, oligomycin has been shown to deplete cellular ATP in the MCF7 cell line [36], but not in MDA-MB-231 [36] or PC-3 cell lines [37].

For the NCI-60 panel, there is no literature concerning the relative contribution of mitochondrial versus glycolytic production in the total cellular ATP pool. According to Evans et al. [33], the highly glycolytic cells have an overexpression of glucose transporter Glut-1 that confers a poor prognosis for a wide variety of solid tumors. To deplete the total ATP content of cell, it would be necessary to inhibit both glycolysis and oxidative phosphorylation. Among the nine cancer cell lines that we tested for ATP production, all proved sensitive for the peptide 9S1R ATP depletion effect, while only four cell lines (U-251, EKVX, COLO 205 and K-562) are sensitive to oligomycin [38]. Our data shows that the dramatic reduction in cellular ATP seen within 3 h of 9S1R incubation is not always concordant with cell death. How this depletion is achieved is still unknown.

We also measured supernatant ATP in nine cancer cell lines, and show that both 9R and 9SR1 deplete supernatant ATP content in only COLO 205, DU-145 and K-562. We hypothesize that the ATP found in the cellular supernatant may have been secreted by the ectopic expression of F_0_F_1_-ATP Synthase, which is relocated to the surface of tumor cells [9]. We note that there is no current drug or chemical, with the exception of anthrax lethal toxin, capable of shutting down all the ATP production in cancer cells. Future work will be needed to exploit the ATP depletion effects of peptides 9R and 9S1R.

### At 25 μM concentration 9R and 9S1R are more lethal to many cancer cell lines than to normal cell lines

We have looked at the vitality of all the NCI-60 cell lines when exposed to 5 μM, 25 μM and 50 μM of each Nullomer-derived peptide (“Additional file 1: Results S1”). We note significant effects against 52 of the 60 cancer cell lines with the incubation of 25 μM 9S1R. This 25 μM dose has no significant effects against the 2 of the 4 normal cell lines we tested: HUVEC (endothelium) and BJ (skin). On a third normal cell line, FHC (intestinal), 25 μM 9S1R resulted in a 74% SI, which is less of a lethal affect than seen for many of the cancer lines tested. While 25 μM 9S1R resulted in an SI of only 21% for RWPE-1 (normal prostate), it should be noted that non-cancerous adult prostate cells may respond quite differently than the early-passage cells we screened in culture. It should also be noted that in our earlier study using PCS normal prostate cells, we found that the cancer line LnCap is more sensitive than normal prostate cells [3] to the Nullomer drugs.

As noted in the results, Nullomer-derived 9R is generally safer for normal cells than 9S1R. At 25 μM, 9R has no effect on HUVEC or BJ normal cell lines; and the effect on the intestinal cell line (FHC) was below our sensitivity threshold of 20% (90% remained viable). Of the four normal cells lines examined, RWPE-1 prostate cells showed the greatest sensitivity to 9R, dropping just below our sensitivity threshold of 20%, (the cells were 79% viable).

## Conclusion

Current cancer therapy suffers from many limitations including drug resistance and toxicity. We have produced novel anti–cancer drugs based on the shortest peptide sequences absent form natural proteins, Nullomers. These pentamer (5-amino acid) sequences combined with five R residues (for cell penetration) are effective against 58 of the 60 cancer cell types in the NCI-60 cancer panel. While the mechanism (s) of their effects require further research, the peptides clearly affect cellular ATP production, have differential effects on various cancer cell types, and are more toxic to many cancers than most normal cell types tested.

No current anti-cancer drug, even those targeting DNA replication or the mitotic apparatus, is effective against cancers derived from all nine organs represented in the NCI-60 panel. However, Nullomer-derived peptides 9S1R and 9R are active against 95 and 81.6%, respectively, of the NCI-60 cancer cell lines. Both peptides are active against dividing and quiescent, drug resistant and drug sensitive, hormone sensitive and hormone resistant, differentiated and non-differentiated, metastatic and non-metastatic cancer cell lines. In addition, both peptides are active against cancer stem cell lines. Therefore, peptides 9S1R and 9R address the problem of *tumor heterogeneity*--a fundamental challenge in cancer treatment. We propose the hypotheses that this broad activity spectrum is due to the fact that both peptides target “*cells of origin of cancer”* [14], these are cells carrying common marker (s) of cancers derived from many organs. Both peptides, especially peptide 9S1R, kill cancer cells by rapidly depleting ATP content. Future studies will evaluate the Nullomer-derived peptides’ effects on invasion ability and metastasis in animal models. Studies that combine the Nullomer-derived peptides with other drugs will examine if depriving ATP-dependent pumps of power, will decrease MDR caused by the efflux of anti-cancer drugs.

## Additional files

Additional file 1: Results 1. Effect of 50 μM, 25 μM and 5 μM Nullomer peptides on cancer cell line growth. Cells (3000-5000 cells/well) were seeded in 96-well plates. After 24 h incubation, peptides 9R, 9S1R and 124R were added to the wells with untreated cells as control. After 48 h exposure to the peptides, cell viability was quantified by the addition of 10 μl of cell counting kit (CCK-8, Dojindo Japan) to each well, which was then incubated for 4 h at 37 °C in a 5% CO2 incubator. After incubation, plates were monitored by a microplate reader (BioTek) at an absorbance of 450 nm. Controls: untreated cells and empty wells. Each panel is a representative of three experiments. (A) Kidney, (B) Prostate, (C) Ovarian, (D) Leukemia Lymphoma, (E) Colon, (F) Melanoma, (G) Breast, (H) CNS, and (I) Lung cancer cell lines. Results are as mean ± SE (standard error) of three different experiments. NS, not significant. *p < 0.05,**p < 0.01,***p < 0.001. (PDF 534 kb)

Additional file 2: Table 1. Survival index (SI) and response categories of the NCI-60 panel. A: SI of NCI-60 cancer cell lines after 48 h with 50 μM peptides 9R, 9S1R and 124R; B: % of cell lines sensitive and resistant to peptides 9R, 9S1R and 124R; C: Peptides 9R, 9S1R and 124R response categories; D: SI and response categories for normal cell lines treated with 9R, 9S1R and 124R. Sensitive lines experience at least 20% decrease in the number of living cells after 48 h treatment. (XLSX 14 kb)

Additional file 3: Table 2. Spectrum activity of peptides 9R, 9S1R and 124R and current anticancer drugs (reference [9]). Comparison of Nullomer derived peptides (9R and 9S1R) sensitivity with current anticancer drugs approved by the FDA. (XLSX 13 kb)

Additional file 4:Results 2. Effect of 50 μM, 25 μM and 5 μM Nullomer peptides on normal cell growth. Cells (3000-5000 cells/well) were seeded in 96-well plates. After 24 h incubation, peptides 9R, 9S1R and 124R were added to the wells, with untreated cells as control. After 48 h exposure to the peptides, cell viability was quantified by the addition of 10 μl of cell counting kit (CCK-8, Dojindo Japan) to each which was then incubated for 4 h at 37 °C in a 5% CO2 incubator. After the incubation, the plates were monitored by a microplate reader (BioTek) at an absorbance of 450 nm. (A) Endothelial cell line, (B) Skin fibroblast, (C) Prostate cell line, (D) Intestinal cell line. Results are as mean ± SE (standard error) of three different experiments. NS, not significant. *p<0.05,**p <0.01, ***p<0.001. (PDF 276 kb)

Additional file 5:Table 3 ATP depletion of cancer and normal cell lines after treatment with 9R, 9S1R and 124R. ATP content: expressed as % of control. Three-hour incubations with 50uM peptide. (XLSX 9 kb)

## Abbreviations

1 M: 1 Molar
ACP: Anti-cancer peptides
ATL: Adult T cell leukemia
ATP: Adenosine triphosphate
BPE: Bovine pituitary extract
CCK-8: Cell counting Kit-8
CCLE: Cancer cell line encyclopedia
CGP: Cancer genome project
CNS: Central nervous system
DT: Doubling time
FBS: Fetal bovine serum
FDA: Food drug administration
MDR: Multi drug resistance
SI: Survival index
USA: United States of America
