# Supplementary material for: The effect of Nullomer-derived peptides 9R, 9S1R and 124R on the NCI-60 panel and normal cell lines
Source: BMC Cancer. 2017 Aug 9;17:533. doi: 10.1186/s12885-017-3514-z (PMC5551024; doi:10.1186/s12885-017-3514-z)
Supplement: Supplementary file 1 — Effect of 50 μM, 25 μM and 5 μM Nullomer peptides on cancer cell line growth. Cells (3000-5000 cells/well) were seeded in 96-well plates. After 24 h incubation, peptides 9R, 9S1R and 124R were added to the wells with untreated cells as control. After 48 h exposure to the peptides, cell viability was quantified by the addition of 10 μl of cell counting kit (CCK-8, Dojindo Japan) to each well, which was then incubated for 4 h at 37 °C in a 5% CO2 incubator. After incubation, plates were monitored by a microplate reader (BioTek) at an absorbance of 450 nm. Controls: untreated cells and empty wells. Each panel is a representative of three experiments. (A) Kidney, (B) Prostate, (C) Ovarian, (D) Leukemia Lymphoma, (E) Colon, (F) Melanoma, (G) Breast, (H) CNS, and (I) Lung cancer cell lines. Results are as mean ± SE (standard error) of three different experiments. NS, not significant. *p < 0.05,**p < 0.01,***p < 0.001. (PDF 534 kb) [file 12885_2017_3514_MOESM1_ESM.pdf]

"Suppl. Results 1"

A. Kidney Cancer Cell Lines: 786-0, CAKI-1, SN12C, RFX 393, TK-10, A 498, UO-31 and ACNH

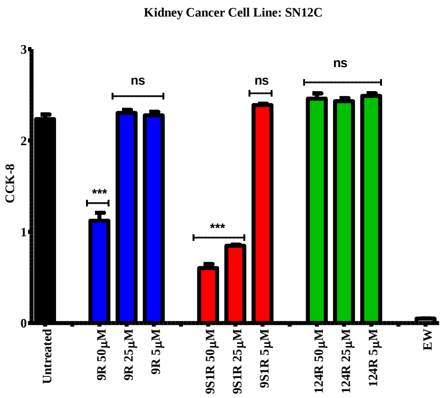

SN12C

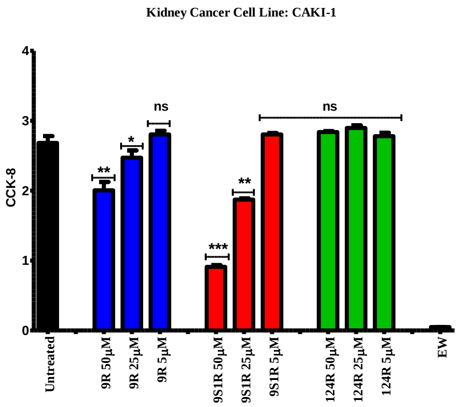

CAKI-1

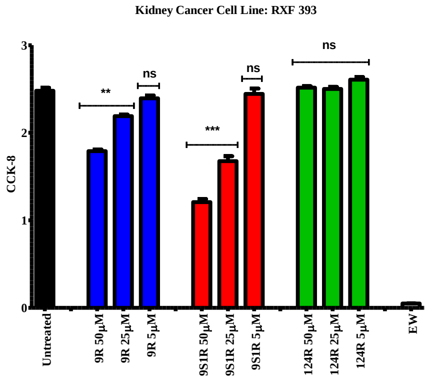

RFX 393

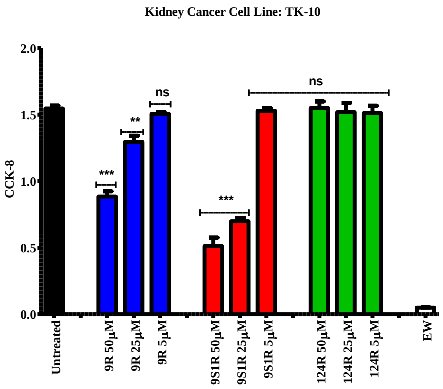

TK-10

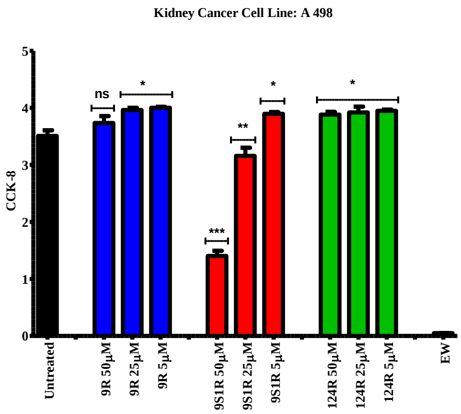

A 498

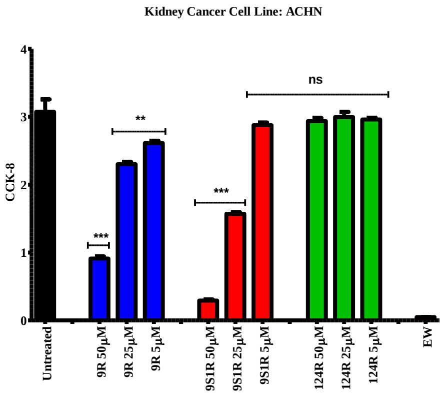

ACNH

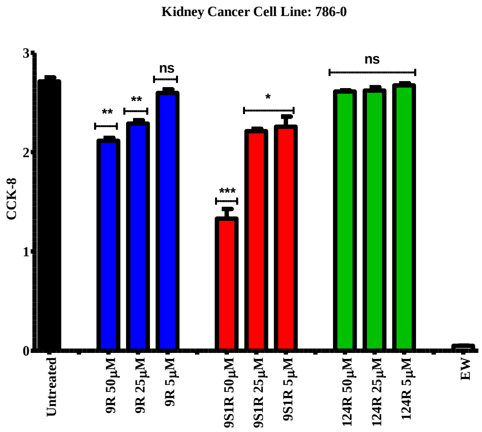

786-0

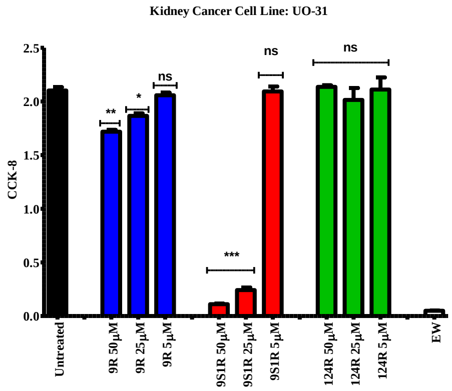

UO-31

B. Prostate Cancer Cell Lines: PC-3, DU-145

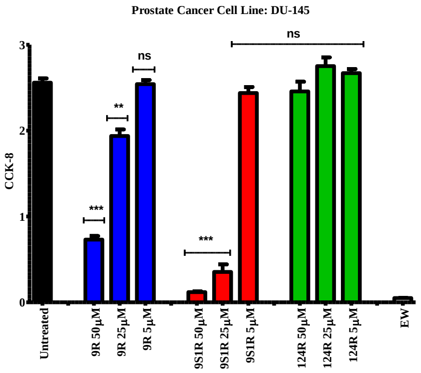

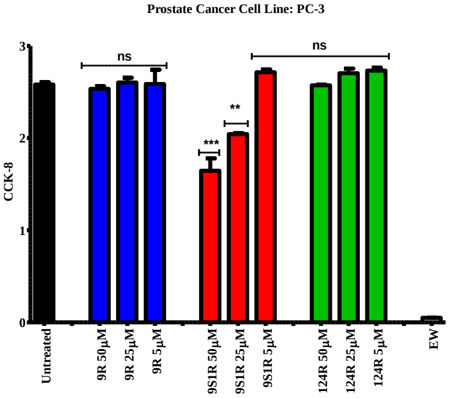

DU-145 **PC-3**

C. Ovarian Cancer Cell Lines: OVCAR-8, NCI/ADR-RES, SK-OV-3, OVCAR-3, OVCAR-4, OVCAR-5 and IGR-OV1

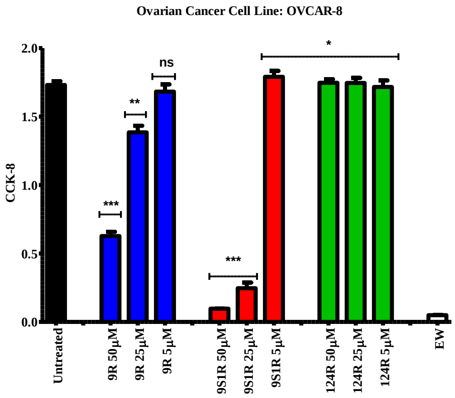

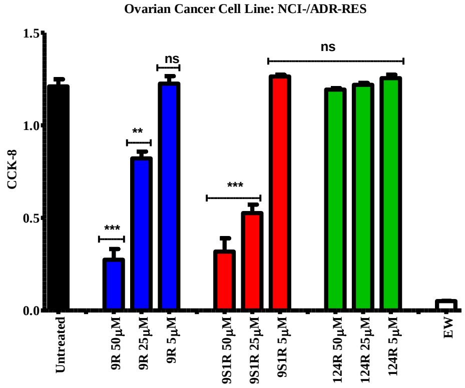

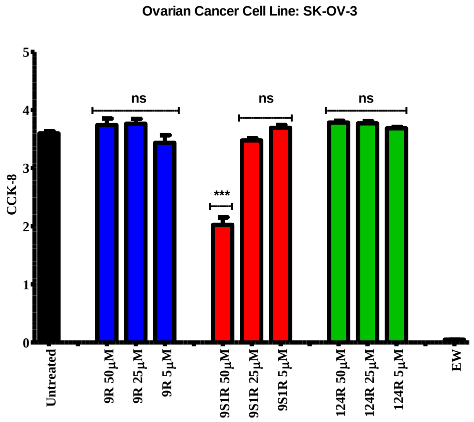

OVCAR-8 **NCI/ADR-RES** **SK-OV-3**

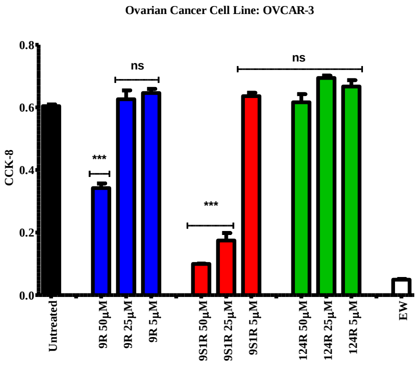

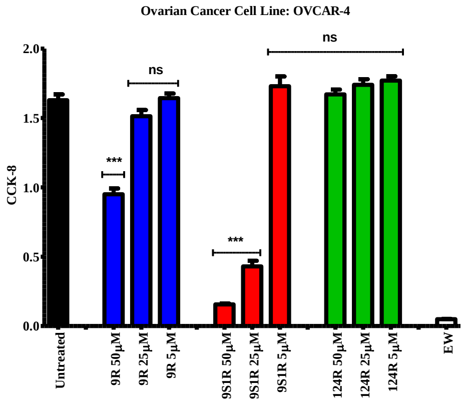

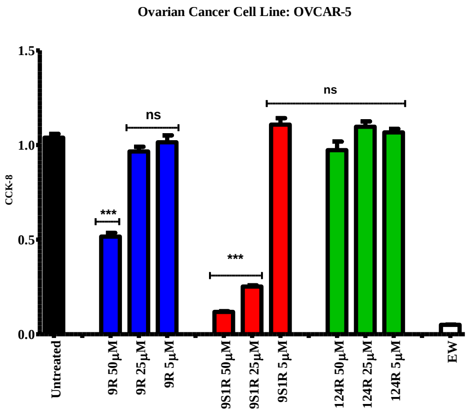

OVCAR-3 **OVCAR-4** **OVCAR-5**

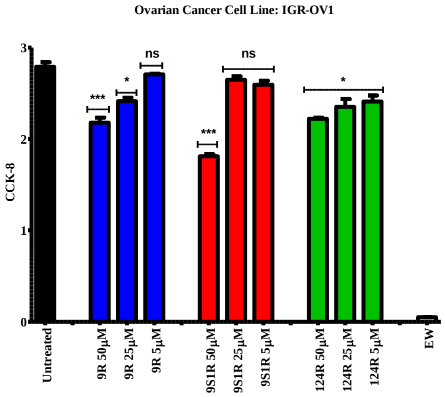

IGR-OV1

D. Leukemia Lymphoma Cell Lines: K-562, SR, CCRF-CEM, RPMI 8226, HL-60 (TB) and MOLT-4

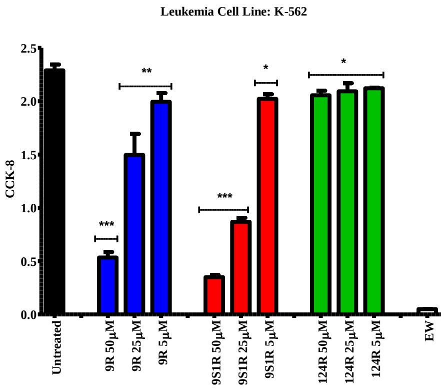

K-562

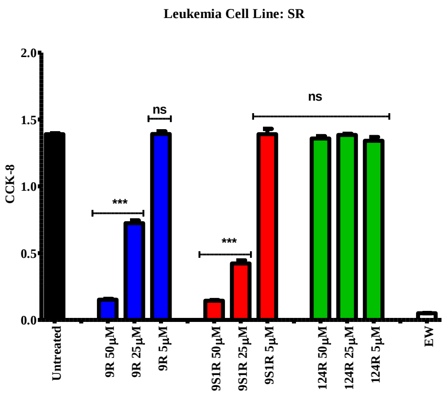

SR

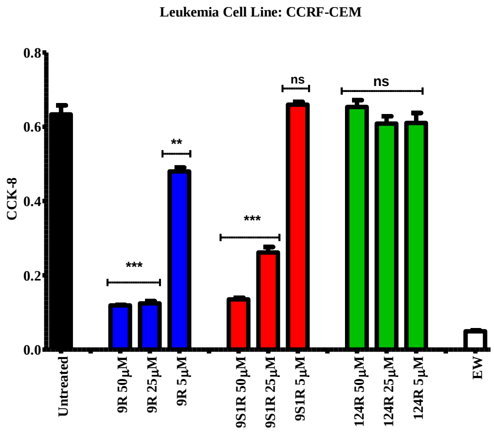

CCRF-CEM

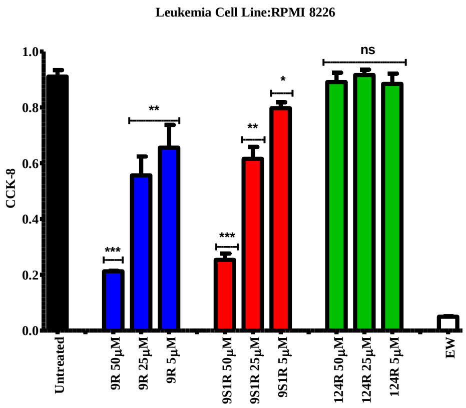

RPMI 8226

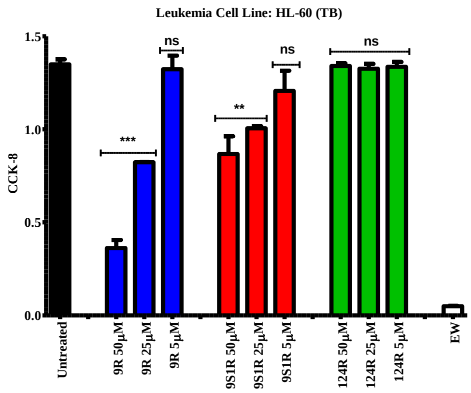

HL-60 (TB)

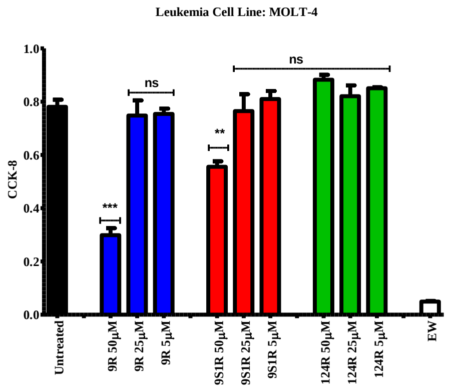

MOLT-4

E. Colon Cancer Cell Lines: KM12, HCC 2998, SW-620, HT-29, HCT-15, HCT-116 and COLO 205

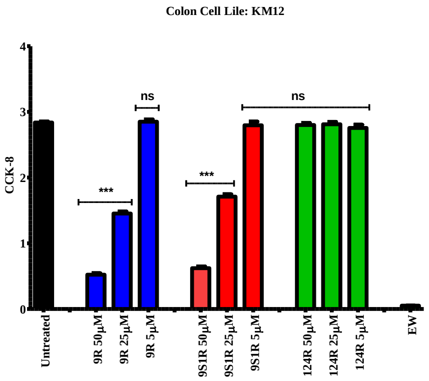

KM12

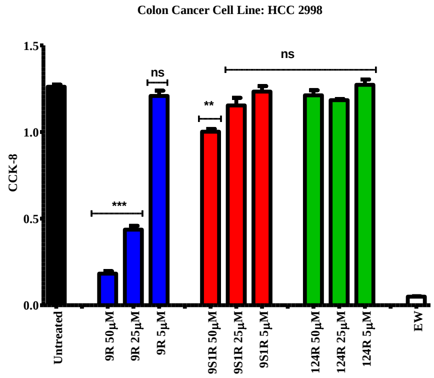

HCC 2998

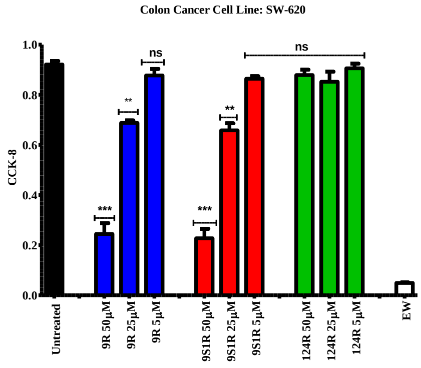

SW-620

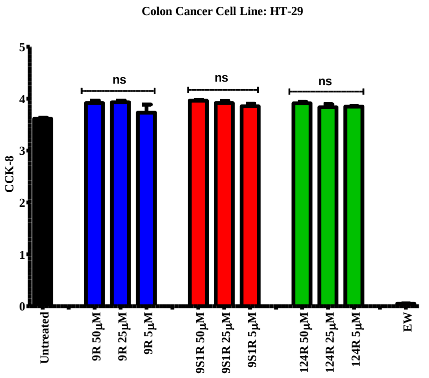

HT-29

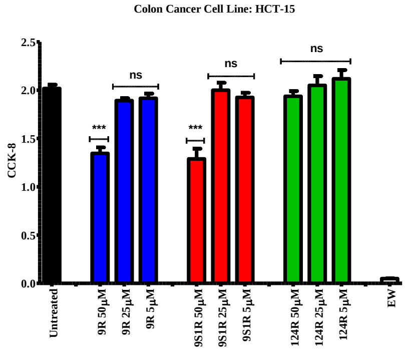

HCT-15

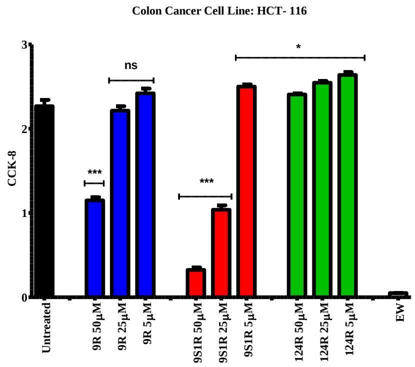

HCT-116

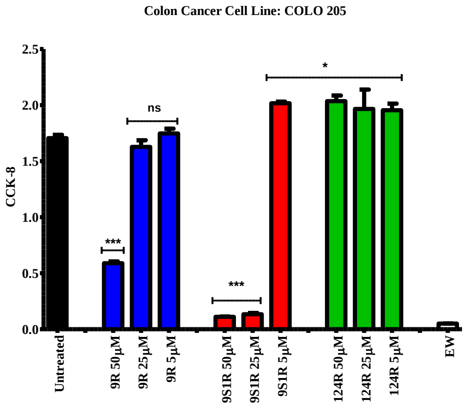

COLO 205

F. Melanoma Cell Lines: UACC-62, SK-MEL-2, SK-MEL-28, M14, LOX-IMVI, UACC-257, SK-MEL-5, MDA-MB-435 and MALME-3M

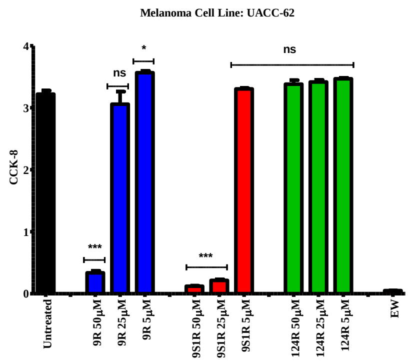

UACC-62

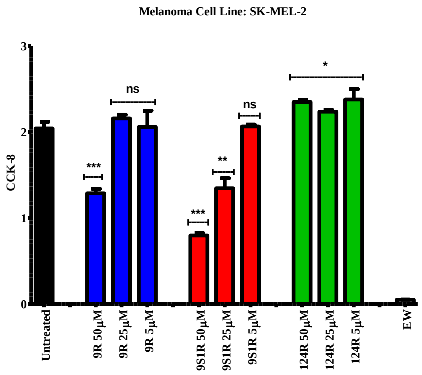

SK-MEL-2

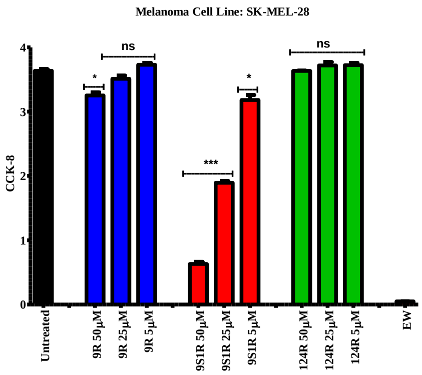

SK-MEL-28

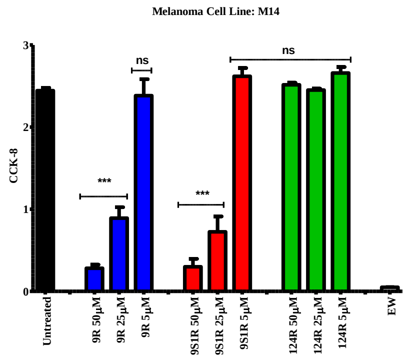

M14

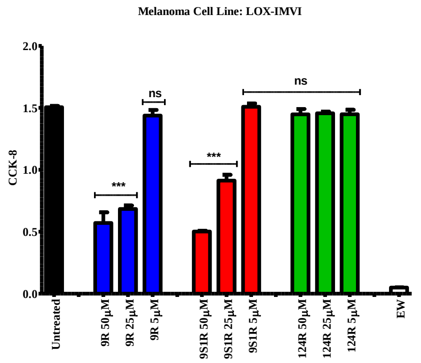

LOX-IMVI

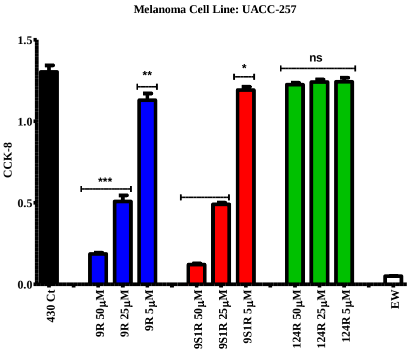

UACC-257

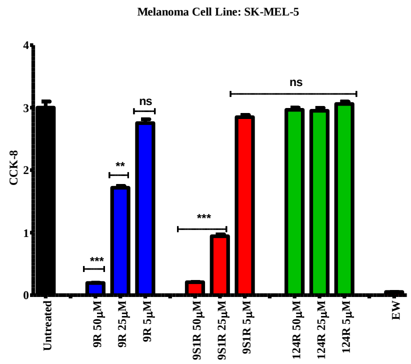

SK-MEL-5

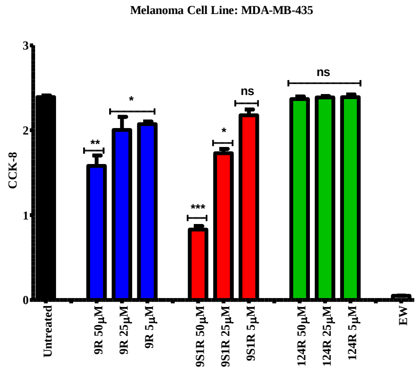

MDA-MB-435

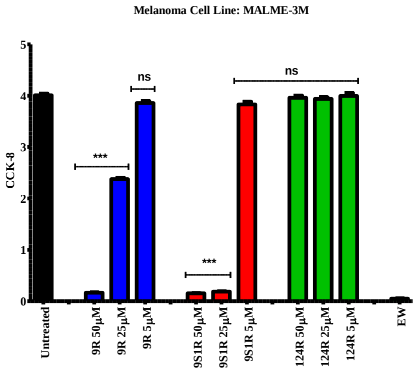

MALME-3M

G. Breast Cancer Cell Lines: T-47D, BT-549, HS-578T, MDA-MB-468, MDA-MB-231 and MCF-7

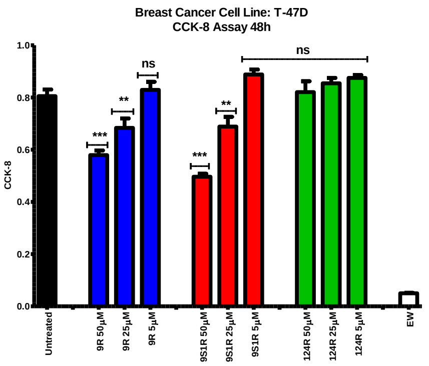

T-47D

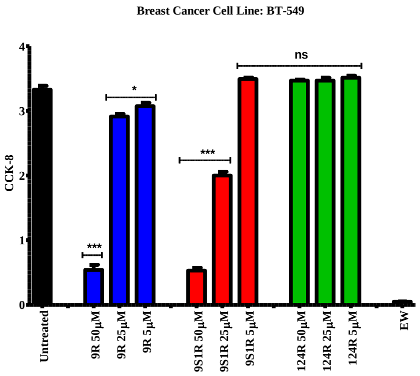

BT-549

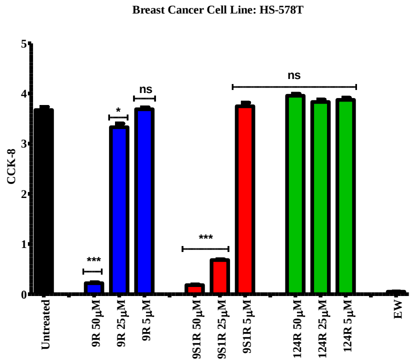

HS-578T

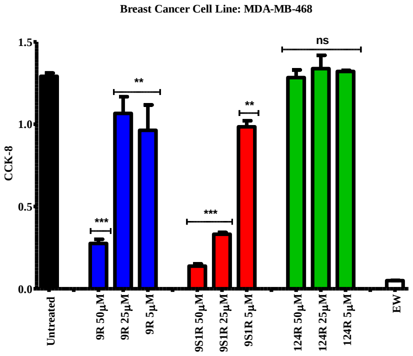

MDA-MB-468

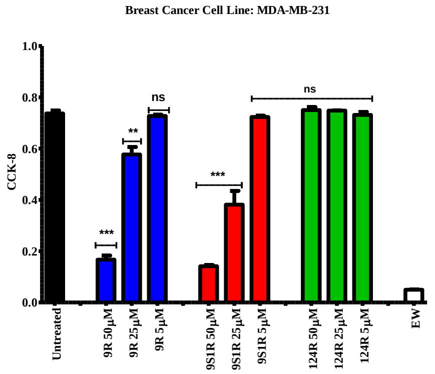

MDA-MB-231

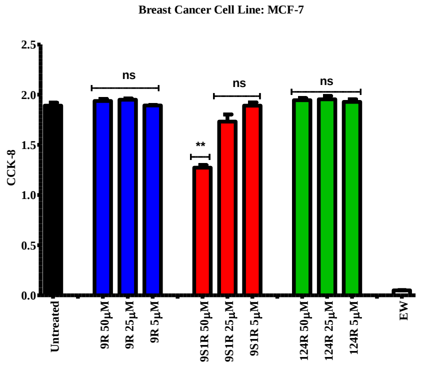

MCF-7

H. CNS Cancer Cell Lines: U251, SNB-75, SNB-19, SF-539, SF-295 and SF-268

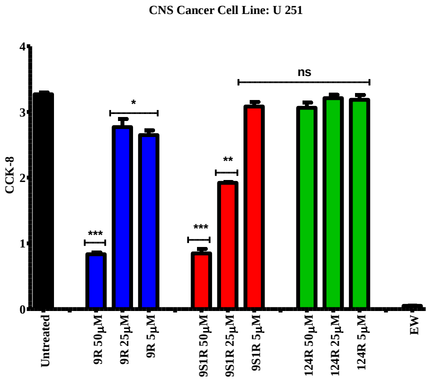

U251

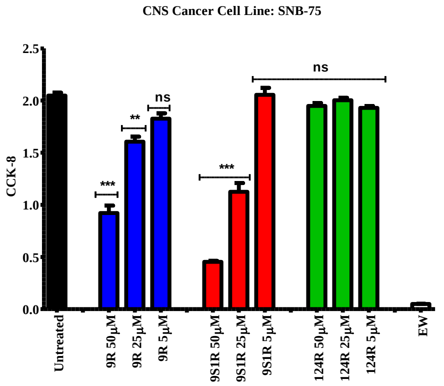

SNB-75

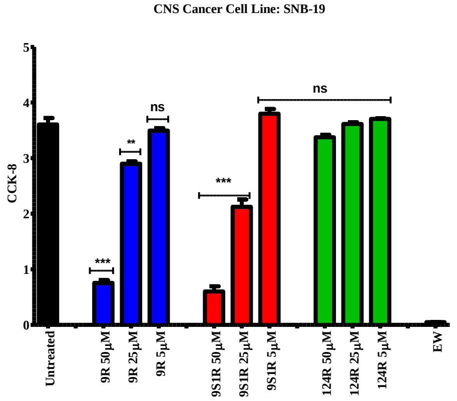

SNB-19

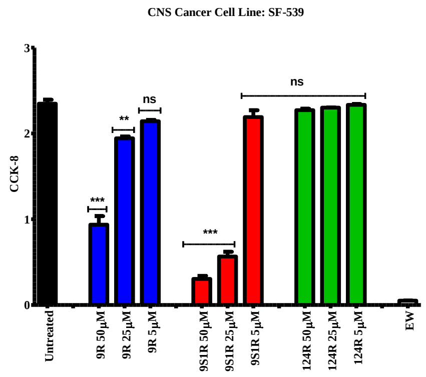

SF-539

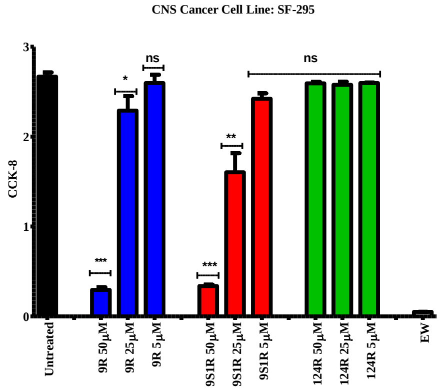

SF-295

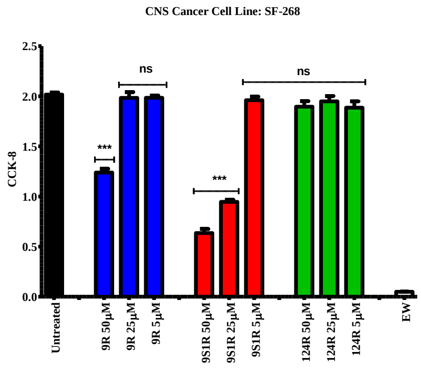

SF-268

I. Lung Cancer Cell Lines: NCI-H23, NC-H226, NCH-H522, A549 ATCC, EKVX, NCI-H460, HOP 62, HOP 92 and NCI-H322M

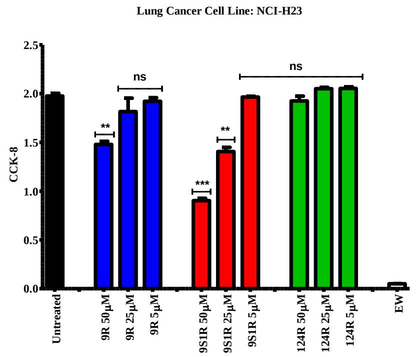

NCI-H23

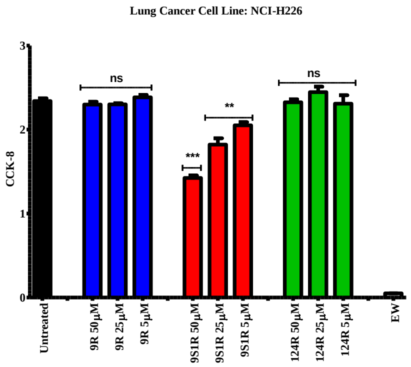

NC-H226

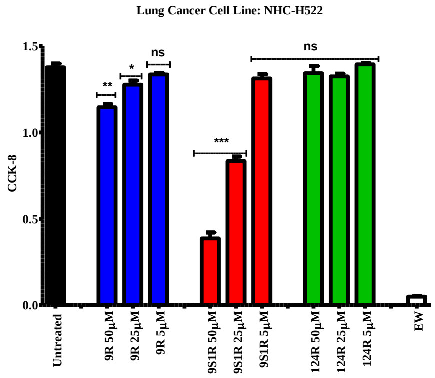

NCH-H522

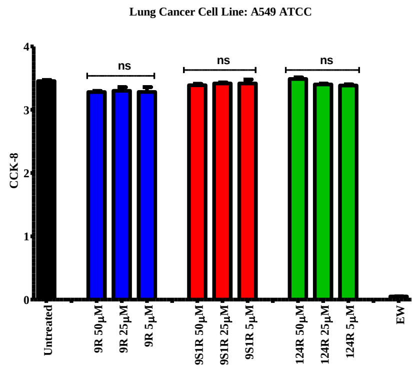

A549 ATCC

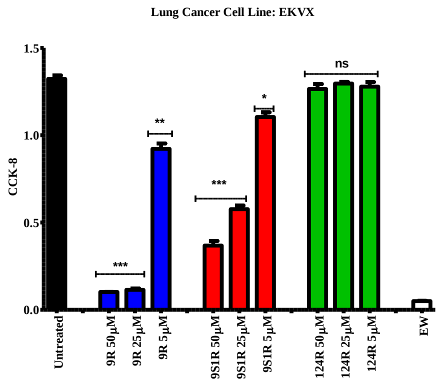

EKVX

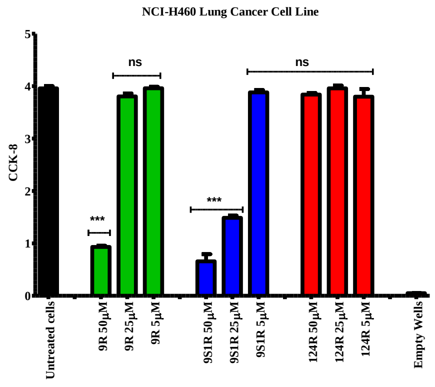

NCI-H460

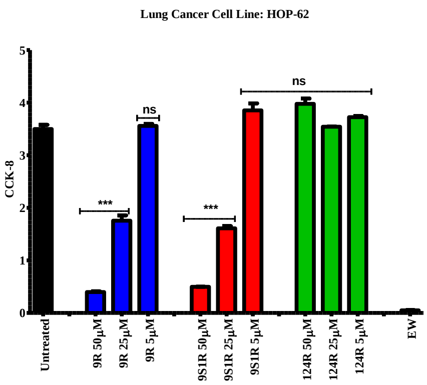

HOP 62

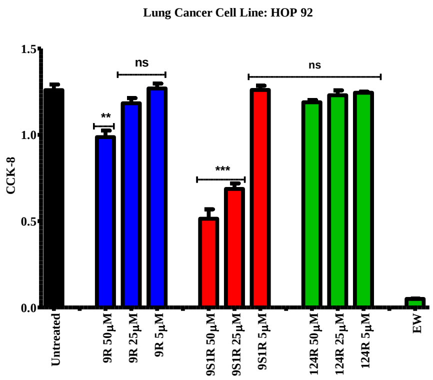

HOP 92

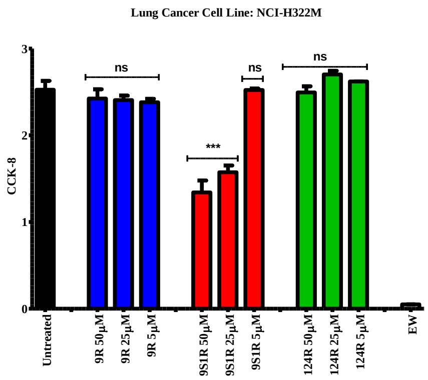

NCI-H322M
