# Supplementary figures and images for: The effect of Nullomer-derived peptides 9R, 9S1R and 124R on the NCI-60 panel and normal cell lines
Source: BMC Cancer. 2017 Aug 9;17:533. doi: 10.1186/s12885-017-3514-z (PMC5551024; doi:10.1186/s12885-017-3514-z)

“Suppl. Results 2”

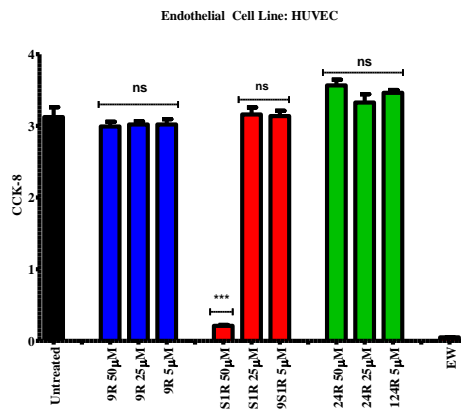

(A) HUVEC

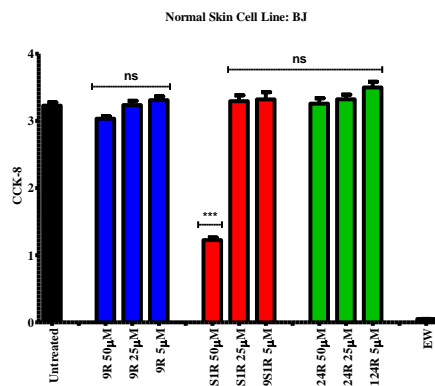

(B) BJ

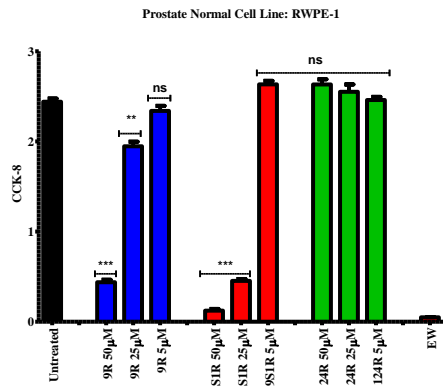

(C) RPEW -1

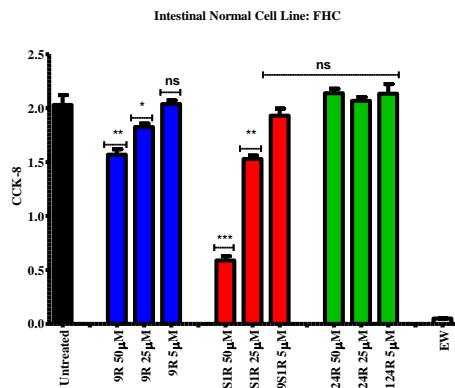

(D) FHC

Supplement: Supplementary file 4 — Effect of 50 μM, 25 μM and 5 μM Nullomer peptides on normal cell growth. Cells (3000-5000 cells/well) were seeded in 96-well plates. After 24 h incubation, peptides 9R, 9S1R and 124R were added to the wells, with untreated cells as control. After 48 h exposure to the peptides, cell viability was quantified by the addition of 10 μl of cell counting kit (CCK-8, Dojindo Japan) to each which was then incubated for 4 h at 37 °C in a 5% CO2 incubator. After the incubation, the plates were monitored by a microplate reader (BioTek) at an absorbance of 450 nm. (A) Endothelial cell line, (B) Skin fibroblast, (C) Prostate cell line, (D) Intestinal cell line. Results are as mean ± SE (standard error) of three different experiments. NS, not significant. *p<0.05,**p <0.01, ***p<0.001. (PDF 276 kb) [file 12885_2017_3514_MOESM4_ESM.pdf]
